# Developmental Disturbance in Premolars After Intraligamental Anesthesia Using Computer-Controlled Local Anesthesia Delivery System: An-Eight-Years Follow-Up Study in Children

**DOI:** 10.7759/cureus.50985

**Published:** 2023-12-23

**Authors:** Khlood Baghlaf, Sara M Bagher, Rana A Alamoudi, Ehda Falemban, Hanin Badiab, Heba Sabbagh

**Affiliations:** 1 Pediatric Dentistry, King Abdulaziz University, Jeddah, SAU

**Keywords:** succedanous, dental bud, sta, high-pressure anesthesia, hypoplasia

## Abstract

Aim

This study aimed to evaluate the prevalence of developmental disturbances in permanent second premolars in which their tooth buds were exposed to mandibular intraligamental anesthesia (ILA) using a computer-controlled local anesthetic delivery system (CCLAD).

Materials and methods

This was a longitudinal follow-up study conducted in a previous randomized clinical trial (RCT). In the previous RCT, a total of 91 children were included (61 control and 30 cases). A structured form was created that contained details about the date of birth, age, and sex at which the participants received local anesthesia and the type of local anesthesia administered (ILA using CCLADS, traditional inferior alveolar nerve block [IANB], and IANB using CCLADS). A history of post-treatment abscess, retreatment, and post-treatment extraction was documented in both groups. Descriptive statistics, including frequency and percentage, and additionally, the chi-square test and Fisher's exact test were used to compare ILA and IANB.

Results

Forty of the 91 children attended follow-up visits. Only two children had developmental defects: one child who received traditional IANB had a demarcated white opacity (this patient had a history of dental abscess), and another who received ILA using CCLADS showed hypoplasia on his permanent premolar. No significant association was found between the type of anesthesia and the presence of developmental defects.

Conclusion

The slow administration of ILA delivered by CCLADS in the primary teeth does not increase the chances of developmental disturbances or damage to the corresponding permanent tooth bud.

## Introduction

Intraligamentary anesthesia (ILA) is an easy alternative for the mandibular block that provides a short duration of anesthesia and requires a small dose of anesthetic fluid using high pressure [1]. ILA has been used in primary molars where regional block anesthesia has previously failed [2]. Therefore, it interferes with the permanent tooth bud and causes postoperative pain and trauma to the periodontal tissue in the area of injection [2].

Recently, a computer-controlled local anesthetic delivery system (CCLAD) has been introduced to reduce pain, in which the anesthetic solution diffuses to the tissue at a controlled rate [2-4]. CCLAD has several advantages, including an integrated dynamic pressure sensing (DPS) technology for measuring real-time pressure. Additionally, the single-tooth anesthesia (STA) [4] system uses slow-to-moderate pressures and a computer-controlled rate of flow during administration.

CCLAD has been implemented on primary teeth and primary molars [2]. Several clinical trials have investigated the efficiency and the efficacy of the ILA using CCLAD in reducing pain-related behaviors during the injection compared to the inferior alveolar nerve block (IANB) [5-9]. However, limited attention has been paid to the effects of ILA using CCLAD on the underlying permanent tooth bud [10].

To our knowledge, only one study by Ashkenazi et al. (2010) reported the use of ILA using CCLAD on primary second molars during their developmental stage and concluded that ILA does not increase the risk of developmental disturbances to the underlying permanent dental bud [10]. Therefore, the purpose of our study was to evaluate the prevalence of developmental disturbances in second premolars in which their buds were exposed to mandibular ILA using CCLAD. We hypothesized that using ILA with CCLAD on the mandibular second primary molars was not associated with an increased prevalence of developmental disturbances in their permanent succedaneous teeth.

## Materials and methods

Study design and ethical approval

A longitudinal follow-up study of a previously randomized clinical trial (RCT) was performed in accordance with the principles outlined in the Declaration of Helsinki.

The King Abdulaziz University Faculty of Dentistry Research (KAUFD) Ethics Committee approved all procedures described in the research study documents in 2022 (approval no. 122-10-22).

Participant selection

The participants included in the study also participated in previously published RCTs to compare the clinical effectiveness of ILA using the CCLADS [11] and its efficiency in decreasing pain and discomfort in comparison to traditional IANB and IANB using CCLADS [8]. All parents signed the consent forms. This RCT study included a total of 91 children from five to nine years old diagnosed with at least one mandibular second primary molar that was carious and required treatment and local anesthesia administration at KAUDH in Jeddah, Saudi Arabia, between November 2012 and April 2014. Thirty children received ILA (cases) using CCLADS, and 61 (controls) received either traditional IANB or IANB using CCLADS.

In the follow-up study, a total of 40 children came to the follow-up visit (31 control and nine cases). The follow-up study was conducted from March 2023 to June 2023.

Sample size and sampling technique

It was expected that 50% attrition of the sample would be enough to provide a comparison after the eight to nine-year follow-up period. Therefore, the expected sample size was 15 participants in the case group and 30 in the control group.

Study technique

The children were identified by a trained general dentist in March 2023 using the R4 electronic filing system at KAUDH. The parents/guardians of the identified children were contacted by phone by trained general dentists, and thorough explanations of the research's purpose, risks, advantages, and drawbacks were provided. All parents who agreed to participate in the study signed the consent form.

Appointments were scheduled for those who agreed to participate. At the scheduled appointment, documentation of the date of birth, sex, age at which the participants received local anesthesia, and the type of local anesthesia administered (ILA using CCLADS, traditional IANB, or IANB using CCLADS) was provided to the second primary mandibular molar on one side. The history of post-treatment abscess, retreatment, and post-treatment extraction in both groups was recorded. All the data were recorded by a single trained general dentist.

Clinical examination

The participants were clinically examined independently by two trained and calibrated general dentists. The erupted permanent successors of previously treated and anesthetized primary second molars by ILA using CCLADS, traditional IANB, or IANB using CCLADS were examined. The Clarkson and O'Mullane classification was used to record the type of developmental disturbance that was found in the mandibular premolars while meeting the following criteria: (a) type of defect, which could be normal, demarcated opacity (white/cream or yellow/brown), diffuse opacity, hypoplasia, and others; (b) the extent of defect: <1⁄3, 1⁄3-2⁄3, and ≥2⁄3; (c) the location of the defect: confined to either the gingival half, incisal half, occlusal, or cuspal; and (d) size of the developmental disturbances: <2 mm^2^, 2-4 mm^2^, and >4 mm^2^. We did not report this in the results section.

For the calibration, 50 slides of teeth with clinical developmental disturbances were provided to each examiner. Each slide was independently evaluated and assessed by each examiner. In cases of disagreement, a third trained and calibrated examiner was asked to perform the examination and evaluate the tooth. Disagreements were resolved by discussion to reach a unanimous decision. An agreement value of 0.9 was considered acceptable.

Statistical analysis

Data analysis was performed with SPSS Statistics for Windows, version 16 (SPSS Inc., US). The standard deviation and mean were used to demonstrate the numerical data. Percentages and frequencies were used to demonstrate quantitative data. Inter- and intra-examiner reliabilities were assessed using the interclass correlation (ICC) test. When comparing the two groups, Fisher's exact test and the chi-square test were used to examine differences. Statistical significance was set at p<0.05.

## Results

An agreement of 85.6% was recorded between the two trained examiners. However, the reliabilities recorded by each of the two examiners were 88.3% and 86.0%, respectively.

At the baseline, 91 children with a mean age of 15.2±1.5 years previously participated in the published RCT to assess the clinical effectiveness and efficiency of ILA using CCLADS [11]. A total of 40 children out of the original 91, with a mean age of 14.94±1.59, were present at the follow-up visit. The response rate was 44% (50% for IANB and 30% for ILA). The mean age and distribution of gender were similar among children participating at the baseline and follow-up (Table 1). The groups that underwent ILA and IANB had similar distributions of age and pulpotomy failure rates.

**Table 1:** Distribution of the sample according to gender and type of anesthetic technique in the baseline and follow-up IANB - inferior alveolar nerve block using both Wand and traditional techniques; ILA - intraligamentary anesthesia using Wand technique * p-value comparing the baseline and the follow-up

Variables		Baseline	Follow-up	p-value*
Sex, n (%)	Male	39 (42.9)	17 (42.5)	0.97
	Female	52 (57.1)	23 (57.5)	
Anesthetic technique, n (%)	IANB	61 (67.0)	31 (77.5)	0.23
	IL	30 (33.0)	9 (22.5)	
Total		91 (100)	40 (100)	

The appendix shows the distribution of the samples according to pulpotomy post-treatment failures. There were five cases (12.5%) that had failures after pulpotomy treatment: two (22.2%) in the ILA group and three (9.7%) among the IANB group, with no statistically significant differences (p=0.247).

Table 2 shows the association between clinical defects in the permanent successors and the type of anesthesia administered to the treated primary second molars. However, the patient who presented with a demarcated opacity had a history of dental abscesses.

**Table 2:** The association between clinical defect in the permanent successors and the type of anesthesia administered to the treated primary second molar IANB - inferior alveolar nerve block using both Wand and traditional techniques; ILA - intraligamentary anesthesia using Wand technique ^a^ demarcated opacity; ^b^ hypoplasia

Clinical presentation of the permanent successors		Type of anesthesia		Total	p-value
		ILA n (%)	IANB n (%)		
Enamel defect	Yes	1 (11.1)^a^	1 (3.2)^b^	2 (5)	0.34
	No	8 (88.88)	30 (96.8)	38 (95)	
Total		9	31	40 (100)	

At the follow-up, 38 (95%) of the permanent successors were normal; one child presented with enamel hypoplasia (who received ILA by CCLADS) and one with demarcated white opacity (who received traditional IANB). The type of anesthesia administered was not significantly associated with the development of a post-treatment abscess (p=0.34).

## Discussion

This was a longitudinal follow-up study of a previous randomized clinical trial (RCT) aimed at assessing the association between ILA and developmental disturbances in the second premolars. The clinical efficiency of ILA has been demonstrated and proven in multiple studies [5-8]. This method of dental anesthesia is effective for all procedures usually performed with local infiltration and/or nerve blocks, such as pulpal therapies, cavity preparations, and extractions [4]. One study concluded that in contrast to traditional IANB, ILA with CCLADS was associated with significantly less pain perception [11]. 

When utilizing conventional ILA in the primary teeth of young children, the side effects of high-pressure anesthesia are considered a possible threat to the permanent successor tooth bud [12,13]. High-pressure injections using a traditional syringe may harm the underlying permanent dental buds when anesthetizing the primary molars [8]. If ILA is administered with a high-pressure syringe, the periodontal tissue may be damaged, which prolongs postoperative pain [3,14].

The long-term effects of ILA delivered by CCLADS have not been extensively investigated; in the present study, the effect of ILA delivered by CCLADS was clinically evaluated over the long term (eight years). This study helps pediatric dentists use the ILA with CCLADS without having to consider or worry about the consequences of this technique on the dental bud of the permanent successor. Contrary to the high pressure applied by conventional ILA, slow administration of the local anesthetic solution by CCLADS is predicted to not harm the periodontal apparatus [14].

Only one longitudinal study was conducted in 2010 by Ashkenazi et al. to investigate the effect of computerized delivery of ILA on the permanent successor tooth buds. Similar to our findings, this study showed that ILA using CCLADS does not put the underlying dental buds at risk of developmental disturbances [10].

The control group in the present study included 61 children who received either traditional IANB or IANB using CCLADS, while 30 children who received ILA using CCLADS served as the case group. However, there were 51 (56%) children were lost to the follow-up appointment, which was performed eight years after the anesthesia was administered. This was a limitation of this longitudinal study.

After completion of the recall visits and after analyzing the data, the total results of 40 children showed that two out of 40 patients presented with developmental disturbances - one presented with hypoplasia, and one had a demarcated white opacity. The hypoplasia found in the first patient may not necessarily have been connected to the type of anesthesia used and could not be attributed to dental caries or the type of treatment that the patient received, such as pulpal therapy and stainless steel crowns. However, there was no significant association between the type of anesthesia administered and the development of a post-treatment abscess, the need for retreatment or post-treatment extraction, and the development of clinical defects in the permanent successor. A longitudinal study assessed the association between dental caries in primary molars and enamel defects in premolars and found that permanent teeth were more likely to have a demarcated defect or any defect if there had been caries in the primary precursor by the age of five [14].

The demarcated white opacity found in the permanent tooth of the second patient, in which the related primary tooth had a dental abscess, the previously mentioned defect could be related to damage caused by the dental abscess. Previous studies have found that pulp necrosis of primary teeth and periapical infection can have consequences for successors, including white or yellow opacity and enamel hypoplasia [14,15]. It is worth noting that both patients were seven years old at the time of anesthesia administration, which corresponds to the dental age after completion of the mandibular premolar crown [16].

Despite the fact that ILA using CCLADS does not cause any potential damage to the underlying dental bud of patients aged ≥5 years, regular follow-up care is advised to be provided for the children that receive ILA using CCLADS to anesthetize their primary molars to eliminate any developmental defect in their premolars [10]. Additionally, there is a close relationship between the primary tooth root and the premolar tooth, but it varies based on the child's age and development [17].

In primary dentition, this technique is frequently avoided by dentists because of concerns regarding how it might affect the development of the corresponding permanent tooth buds. With a more thorough investigation, pediatric dentists will be able to safely utilize ILA using CCLADS without being concerned about the adverse effects of this method on the developing successor tooth bud in patients five to nine years old.

This longitudinal study showed no association between ILA and developmental disturbances in second premolars; however, it had some limitations, including sample loss/patient dropouts for any reason and facilities to accomplish the follow-up visits at the proposed time. Additionally, delayed and ectopic eruptions were not assessed. Dental abscesses of the primary teeth and trauma were assessed based on patient files and patient recall.

## Conclusions

Slowly administered ILA delivered by CCLADS to the primary teeth does not increase the chances of developing any disturbances or damage to the corresponding permanent tooth bud, as the pressure applied during injection is controlled. Thus, using ILA with CCLADS when treating children aged ≥5 years could be an effective and safe alternative to traditional IANB without the risk of causing damage to the underlying permanent premolar. 

## Appendices

**Table 3:** The association between the history of posttreatment abscess, retreatment, and post-treatment extraction, and the type of anesthesia administered to the treated primary second molar IANB - inferior alveolar nerve block using both Wand and traditional techniques; ILA - intraligamentary anesthesia using Wand technique * p-value comparing ILA and IANB

Variables		Type of anesthesia		Total	p-value*
		ILA n (%)	IANB n (%)		
Age (years), mean ± SD		14.96 ± 1.62	14.84 ± 1.58	14.94 ± 1.59	0.85
Pulpotomy failures	Yes	2 (22.2)	3 (9.7)	5 (12.5)	0.247
	No	7 (77.8)	28 (90)	35(87.5)	
Post-treatment abscess	Yes	2 (22.22%)	2 (6.5)	4(10)	0.165
	No	7 (77.77)	29 (93.5)	36 (90)	
Required re-treatment	Yes	0 (0)	1 (3.2)	1 (2.5)	1
	No	9 (100)	30 (96.8)	39 (97.5)	
Required extraction	Yes	2 (22.2)	3 (9.7)	3 (7.5)	0.316
	No	7 (77.8)	28 (90.3)	37 (92.5)	
Total		9	31	40 (100)	
